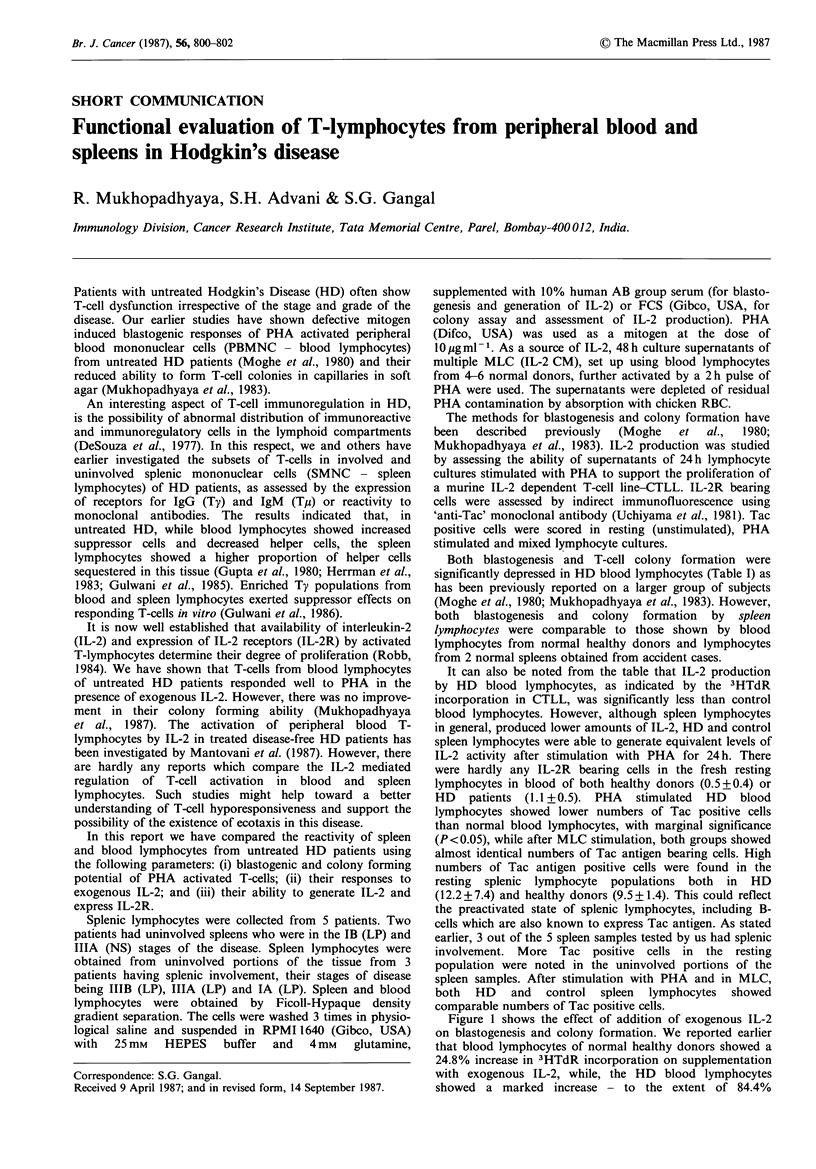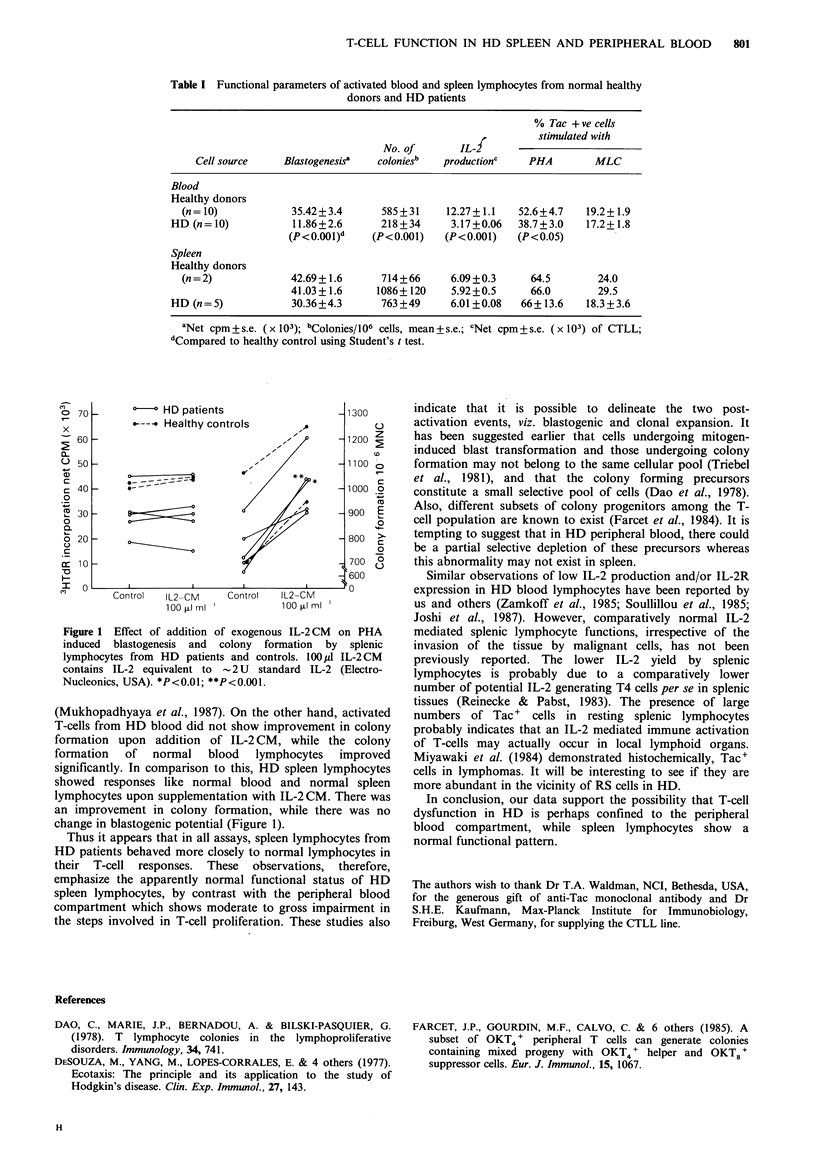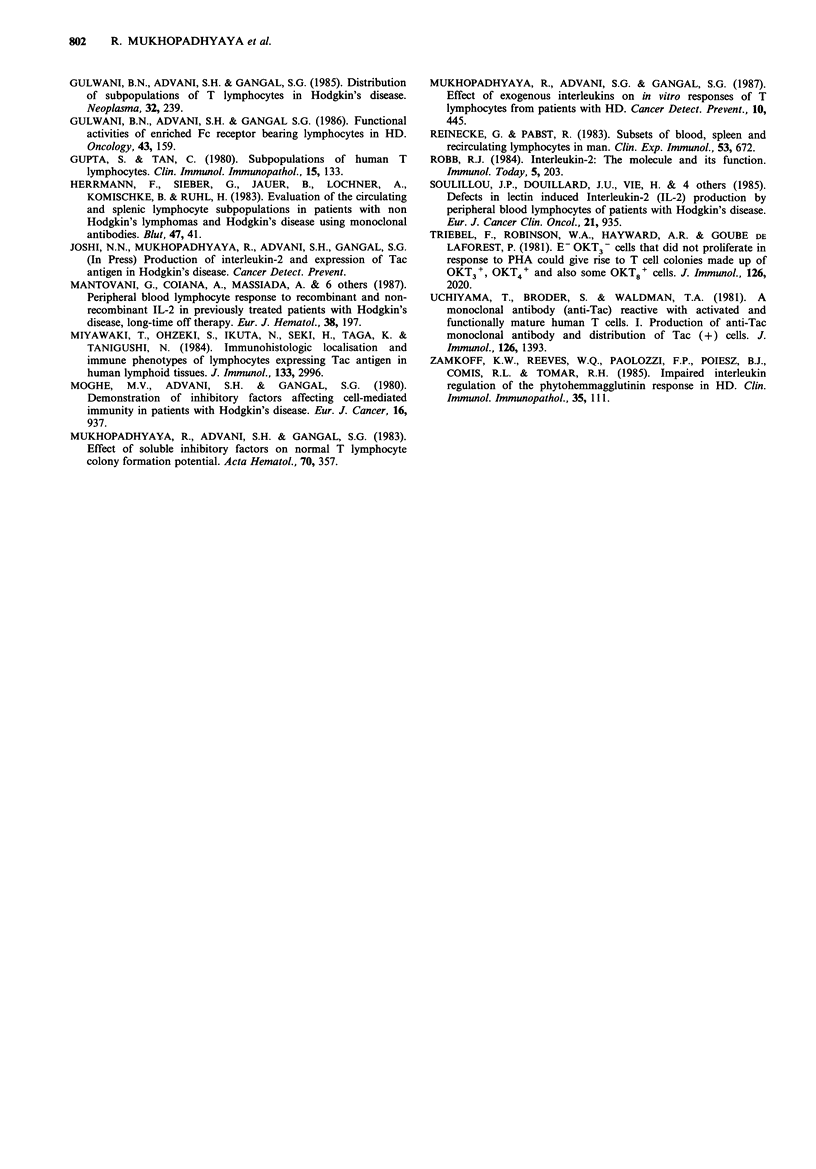# Functional evaluation of T-lymphocytes from peripheral blood and spleens in Hodgkin's disease.

**DOI:** 10.1038/bjc.1987.293

**Published:** 1987-12

**Authors:** R. Mukhopadhyaya, S. H. Advani, S. G. Gangal

**Affiliations:** Cancer Research Institute, Tata Memorial Centre, Parel, Bombay, India.


*Br. J. Cancer* (1987), **56**, 800–802 © The Macmillan Press Ltd., 1987

SHORT COMMUNICATION

### Functional evaluation of T-lymphocytes from peripheral blood and spleens in Hodgkin's disease

R. Mukhopadhyaya, S.H. Advani & S.G. Gangal

*Immunology Division, Cancer Research Institute, Tata Memorial Centre, Parel, Bombay-400012, India.*

Patients with untreated Hodgkin's Disease (HD) often show T-cell dysfunction irrespective of the stage and grade of the disease. Our earlier studies have shown defective mitogen induced blastogenic responses of PHA activated peripheral blood mononuclear cells (PBMNC – blood lymphocytes) from untreated HD patients (Moghe *et al.*, 1980) and their reduced ability to form T-cell colonies in capillaries in soft agar (Mukhopadhyaya *et al.*, 1983).

An interesting aspect of T-cell immunoregulation in HD, is the possibility of abnormal distribution of immunoreactive and immunoregulatory cells in the lymphoid compartments (DeSouza *et al.*, 1977). In this respect, we and others have earlier investigated the subsets of T-cells in involved and uninvolved splenic mononuclear cells (SMNC – spleen lymphocytes) of HD patients, as assessed by the expression of receptors for IgG (Tγ) and IgM (Tμ) or reactivity to monoclonal antibodies. The results indicated that, in untreated HD, while blood lymphocytes showed increased suppressor cells and decreased helper cells, the spleen lymphocytes showed a higher proportion of helper cells sequestered in this tissue (Gupta *et al.*, 1980; Herrman *et al.*, 1983; Gulwani *et al.*, 1985). Enriched Tγ populations from blood and spleen lymphocytes exerted suppressor effects on responding T-cells *in vitro* (Gulwani *et al.*, 1986).

It is now well established that availability of interleukin-2 (IL-2) and expression of IL-2 receptors (IL-2R) by activated T-lymphocytes determine their degree of proliferation (Robb, 1984). We have shown that T-cells from blood lymphocytes of untreated HD patients responded well to PHA in the presence of exogenous IL-2. However, there was no improvement in their colony forming ability (Mukhopadhyaya *et al.*, 1987). The activation of peripheral blood T-lymphocytes by IL-2 in treated disease-free HD patients has been investigated by Mantovani *et al.* (1987). However, there are hardly any reports which compare the IL-2 mediated regulation of T-cell activation in blood and spleen lymphocytes. Such studies might help toward a better understanding of T-cell hyporesponsiveness and support the possibility of the existence of ecotaxis in this disease.

In this report we have compared the reactivity of spleen and blood lymphocytes from untreated HD patients using the following parameters: (i) blastogenic and colony forming potential of PHA activated T-cells; (ii) their responses to exogenous IL-2; and (iii) their ability to generate IL-2 and express IL-2R.

Splenic lymphocytes were collected from 5 patients. Two patients had uninvolved spleens who were in the IB (LP) and IIIA (NS) stages of the disease. Spleen lymphocytes were obtained from uninvolved portions of the tissue from 3 patients having splenic involvement, their stages of disease being IIIB (LP), IIIA (LP) and IA (LP). Spleen and blood lymphocytes were obtained by Ficoll-Hypaque density gradient separation. The cells were washed 3 times in physiological saline and suspended in RPMI 1640 (Gibco, USA) with 25 mM HEPES buffer and 4 mM glutamine, supplemented with 10% human AB group serum (for blastogenesis and generation of IL-2) or FCS (Gibco, USA, for colony assay and assessment of IL-2 production). PHA (Difco, USA) was used as a mitogen at the dose of $10\,\mu g\,ml^{-1}$. As a source of IL-2, 48 h culture supernatants of multiple MLC (IL-2 CM), set up using blood lymphocytes from 4–6 normal donors, further activated by a 2 h pulse of PHA were used. The supernatants were depleted of residual PHA contamination by absorption with chicken RBC.

The methods for blastogenesis and colony formation have been described previously (Moghe *et al.*, 1980; Mukhopadhyaya *et al.*, 1983). IL-2 production was studied by assessing the ability of supernatants of 24 h lymphocyte cultures stimulated with PHA to support the proliferation of a murine IL-2 dependent T-cell line–CTLL. IL-2R bearing cells were assessed by indirect immunofluorescence using 'anti-Tac' monoclonal antibody (Uchiyama *et al.*, 1981). Tac positive cells were scored in resting (unstimulated), PHA stimulated and mixed lymphocyte cultures.

Both blastogenesis and T-cell colony formation were significantly depressed in HD blood lymphocytes (Table I) as has been previously reported on a larger group of subjects (Moghe *et al.*, 1980; Mukhopadhyaya *et al.*, 1983). However, both blastogenesis and colony formation by *spleen lymphocytes* were comparable to those shown by blood lymphocytes from normal healthy donors and lymphocytes from 2 normal spleens obtained from accident cases.

It can also be noted from the table that IL-2 production by HD blood lymphocytes, as indicated by the $^3$HTdR incorporation in CTLL, was significantly less than control blood lymphocytes. However, although spleen lymphocytes in general, produced lower amounts of IL-2, HD and control spleen lymphocytes were able to generate equivalent levels of IL-2 activity after stimulation with PHA for 24 h. There were hardly any IL-2R bearing cells in the fresh resting lymphocytes in blood of both healthy donors ($0.5 \pm 0.4$) or HD patients ($1.1 \pm 0.5$). PHA stimulated HD blood lymphocytes showed lower numbers of Tac positive cells than normal blood lymphocytes, with marginal significance ($P < 0.05$), while after MLC stimulation, both groups showed almost identical numbers of Tac antigen bearing cells. High numbers of Tac antigen positive cells were found in the resting splenic lymphocyte populations both in HD ($12.2 \pm 7.4$) and healthy donors ($9.5 \pm 1.4$). This could reflect the preactivated state of splenic lymphocytes, including B-cells which are also known to express Tac antigen. As stated earlier, 3 out of the 5 spleen samples tested by us had splenic involvement. More Tac positive cells in the resting population were noted in the uninvolved portions of the spleen samples. After stimulation with PHA and in MLC, both HD and control spleen lymphocytes showed comparable numbers of Tac positive cells.

Figure 1 shows the effect of addition of exogenous IL-2 on blastogenesis and colony formation. We reported earlier that blood lymphocytes of normal healthy donors showed a 24.8% increase in $^3$HTdR incorporation on supplementation with exogenous IL-2, while, the HD blood lymphocytes showed a marked increase – to the extent of 84.4%

Correspondence: S.G. Gangal.
Received 9 April 1987; and in revised form, 14 September 1987.

**Table I** Functional parameters of activated blood and spleen lymphocytes from normal healthy donors and HD patients

| *Cell source* | *Blastogenesis*[a] | *No. of colonies*[b] | *IL-2 production*[c] | *% Tac +ve cells stimulated with* *PHA* | *MLC* |
|---|---|---|---|---|---|
| *Blood* | | | | | |
| Healthy donors ($n=10$) | 35.42±3.4 | 585±31 | 12.27±1.1 | 52.6±4.7 | 19.2±1.9 |
| HD ($n=10$) | 11.86±2.6 | 218±34 | 3.17±0.06 | 38.7±3.0 | 17.2±1.8 |
| | ($P<0.001$)[d] | ($P<0.001$) | ($P<0.001$) | ($P<0.05$) | |
| *Spleen* | | | | | |
| Healthy donors ($n=2$) | 42.69±1.6 | 714±66 | 6.09±0.3 | 64.5 | 24.0 |
| | 41.03±1.6 | 1086±120 | 5.92±0.5 | 66.0 | 29.5 |
| HD ($n=5$) | 30.36±4.3 | 763±49 | 6.01±0.08 | 66±13.6 | 18.3±3.6 |

[a]Net cpm±s.e. (×10³); [b]Colonies/10⁶ cells, mean±s.e.; [c]Net cpm±s.e. (×10³) of CTLL; [d]Compared to healthy control using Student's $t$ test.

**Figure 1** Effect of addition of exogenous IL-2CM on PHA induced blastogenesis and colony formation by splenic lymphocytes from HD patients and controls. 100 µl IL-2CM contains IL-2 equivalent to ~2U standard IL-2 (Electro-Nucleonics, USA). *$P<0.01$; **$P<0.001$.

(Mukhopadhyaya *et al.*, 1987). On the other hand, activated T-cells from HD blood did not show improvement in colony formation upon addition of IL-2CM, while the colony formation of normal blood lymphocytes improved significantly. In comparison to this, HD spleen lymphocytes showed responses like normal blood and normal spleen lymphocytes upon supplementation with IL-2CM. There was an improvement in colony formation, while there was no change in blastogenic potential (Figure 1).

Thus it appears that in all assays, spleen lymphocytes from HD patients behaved more closely to normal lymphocytes in their T-cell responses. These observations, therefore, emphasize the apparently normal functional status of HD spleen lymphocytes, by contrast with the peripheral blood compartment which shows moderate to gross impairment in the steps involved in T-cell proliferation. These studies also indicate that it is possible to delineate the two post-activation events, *viz.* blastogenic and clonal expansion. It has been suggested earlier that cells undergoing mitogen-induced blast transformation and those undergoing colony formation may not belong to the same cellular pool (Triebel *et al.*, 1981), and that the colony forming precursors constitute a small selective pool of cells (Dao *et al.*, 1978). Also, different subsets of colony progenitors among the T-cell population are known to exist (Farcet *et al.*, 1984). It is tempting to suggest that in HD peripheral blood, there could be a partial selective depletion of these precursors whereas this abnormality may not exist in spleen.

Similar observations of low IL-2 production and/or IL-2R expression in HD blood lymphocytes have been reported by us and others (Zamkoff *et al.*, 1985; Soullillou *et al.*, 1985; Joshi *et al.*, 1987). However, comparatively normal IL-2 mediated splenic lymphocyte functions, irrespective of the invasion of the tissue by malignant cells, has not been previously reported. The lower IL-2 yield by splenic lymphocytes is probably due to a comparatively lower number of potential IL-2 generating T4 cells *per se* in splenic tissues (Reinecke & Pabst, 1983). The presence of large numbers of Tac$^+$ cells in resting splenic lymphocytes probably indicates that an IL-2 mediated immune activation of T-cells may actually occur in local lymphoid organs. Miyawaki *et al.* (1984) demonstrated histochemically, Tac$^+$ cells in lymphomas. It will be interesting to see if they are more abundant in the vicinity of RS cells in HD.

In conclusion, our data support the possibility that T-cell dysfunction in HD is perhaps confined to the peripheral blood compartment, while spleen lymphocytes show a normal functional pattern.

The authors wish to thank Dr T.A. Waldman, NCI, Bethesda, USA, for the generous gift of anti-Tac monoclonal antibody and Dr S.H.E. Kaufmann, Max-Planck Institute for Immunobiology, Freiburg, West Germany, for supplying the CTLL line.

### References

DAO, C., MARIE, J.P., BERNADOU, A. & BILSKI-PASQUIER, G. (1978). T lymphocyte colonies in the lymphoproliferative disorders. *Immunology*, **34**, 741.

DESOUZA, M., YANG, M., LOPES-CORRALES, E. & 4 others (1977). Ecotaxis: The principle and its application to the study of Hodgkin's disease. *Clin. Exp. Immunol.*, **27**, 143.

FARCET, J.P., GOURDIN, M.F., CALVO, C. & 6 others (1985). A subset of $OKT_4^+$ peripheral T cells can generate colonies containing mixed progeny with $OKT_4^+$ helper and $OKT_8^+$ suppressor cells. *Eur. J. Immunol.*, **15**, 1067.

GULWANI, B.N., ADVANI, S.H. & GANGAL, S.G. (1985). Distribution of subpopulations of T lymphocytes in Hodgkin's disease. *Neoplasma*, **32**, 239.

GULWANI, B.N., ADVANI, S.H. & GANGAL S.G. (1986). Functional activities of enriched Fc receptor bearing lymphocytes in HD. *Oncology*, **43**, 159.

GUPTA, S. & TAN, C. (1980). Subpopulations of human T lymphocytes. *Clin. Immunol. Immunopathol.*, **15**, 133.

HERRMANN, F., SIEBER, G., JAUER, B., LOCHNER, A., KOMISCHKE, B. & RUHL, H. (1983). Evaluation of the circulating and splenic lymphocyte subpopulations in patients with non Hodgkin's lymphomas and Hodgkin's disease using monoclonal antibodies. *Blut*, **47**, 41.

JOSHI, N.N., MUKHOPADHYAYA, R., ADVANI, S.H., GANGAL, S.G. (In Press) Production of interleukin-2 and expression of Tac antigen in Hodgkin's disease. *Cancer Detect. Prevent.*

MANTOVANI, G., COIANA, A., MASSIADA, A. & 6 others (1987). Peripheral blood lymphocyte response to recombinant and non-recombinant IL-2 in previously treated patients with Hodgkin's disease, long-time off therapy. *Eur. J. Hematol.*, **38**, 197.

MIYAWAKI, T., OHZEKI, S., IKUTA, N., SEKI, H., TAGA, K. & TANIGUSHI, N. (1984). Immunohistologic localisation and immune phenotypes of lymphocytes expressing Tac antigen in human lymphoid tissues. *J. Immunol.*, **133**, 2996.

MOGHE, M.V., ADVANI, S.H. & GANGAL, S.G. (1980). Demonstration of inhibitory factors affecting cell-mediated immunity in patients with Hodgkin's disease. *Eur. J. Cancer*, **16**, 937.

MUKHOPADHYAYA, R., ADVANI, S.H. & GANGAL, S.G. (1983). Effect of soluble inhibitory factors on normal T lymphocyte colony formation potential. *Acta Hematol.*, **70**, 357.

MUKHOPADHYAYA, R., ADVANI, S.G. & GANGAL, S.G. (1987). Effect of exogenous interleukins on *in vitro* responses of T lymphocytes from patients with HD. *Cancer Detect. Prevent.*, **10**, 445.

REINECKE, G. & PABST, R. (1983). Subsets of blood, spleen and recirculating lymphocytes in man. *Clin. Exp. Immunol.*, **53**, 672.

ROBB, R.J. (1984). Interleukin-2: The molecule and its function. *Immunol. Today*, **5**, 203.

SOULILLOU, J.P., DOUILLARD, J.U., VIE, H. & 4 others (1985). Defects in lectin induced Interleukin-2 (IL-2) production by peripheral blood lymphocytes of patients with Hodgkin's disease. *Eur. J. Cancer Clin. Oncol.*, **21**, 935.

TRIEBEL, F., ROBINSON, W.A., HAYWARD, A.R. & GOUBE DE LAFOREST, P. (1981). $E^-OKT_3^-$ cells that did not proliferate in response to PHA could give rise to T cell colonies made up of $OKT_3^+$, $OKT_4^+$ and also some $OKT_8^+$ cells. *J. Immunol.*, **126**, 2020.

UCHIYAMA, T., BRODER, S. & WALDMAN, T.A. (1981). A monoclonal antibody (anti-Tac) reactive with activated and functionally mature human T cells. I. Production of anti-Tac monoclonal antibody and distribution of Tac (+) cells. *J. Immunol.*, **126**, 1393.

ZAMKOFF, K.W., REEVES, W.Q., PAOLOZZI, F.P., POIESZ, B.J., COMIS, R.L. & TOMAR, R.H. (1985). Impaired interleukin regulation of the phytohemmagglutinin response in HD. *Clin. Immunol. Immunopathol.*, **35**, 111.